# A Defective Interfering Influenza RNA Inhibits Infectious Influenza Virus Replication in Human Respiratory Tract Cells: A Potential New Human Antiviral

**DOI:** 10.3390/v8080237

**Published:** 2016-08-22

**Authors:** Claire M. Smith, Paul D. Scott, Christopher O’Callaghan, Andrew J. Easton, Nigel J. Dimmock

**Affiliations:** 1 Institute of Child Health, University College London, 30 Guilford Street, London WC1N 1EH, UK; c.m.smith@ucl.ac.uk (C.M.S.); c.ocallaghan@ucl.ac.uk (C.O.C.); 2 School of Life Sciences, University of Warwick, Coventry CV4 7AL, UK; paul.scott14@nhs.net (P.D.S.); n.j.dimmock@warwick.ac.uk (N.J.D.)

**Keywords:** defective interfering, influenza virus, antiviral, human respiratory cells

## Abstract

Defective interfering (DI) viruses arise during the replication of influenza A virus and contain a non-infective version of the genome that is able to interfere with the production of infectious virus. In this study we hypothesise that a cloned DI influenza A virus RNA may prevent infection of human respiratory epithelial cells with infection by influenza A. The DI RNA (244/PR8) was derived by a natural deletion process from segment 1 of influenza A/PR/8/34 (H1N1); it comprises 395 nucleotides and is packaged in the DI virion in place of a full-length genome segment 1. Given intranasally, 244/PR8 DI virus protects mice and ferrets from clinical influenza caused by a number of different influenza A subtypes and interferes with production of infectious influenza A virus in cells in culture. However, evidence that DI influenza viruses are active in cells of the human respiratory tract is lacking. Here we show that 244/PR8 DI RNA is replicated by an influenza A challenge virus in human lung diploid fibroblasts, bronchial epithelial cells, and primary nasal basal cells, and that the yield of challenge virus is significantly reduced in a dose-dependent manner indicating that DI influenza virus has potential as a human antiviral.

## 1. Introduction

Influenza virus causes annual epidemics and occasional but devastating pandemics that are associated with considerable morbidity and mortality, particularly in the elderly and in young children [1,2]. Over recent years the threat of influenza viruses, such as H5N1, crossing from their natural avian reservoir to humans and evolving to transmit person-to-person has become a major concern. This threat is confounded by a dearth of new strategies in development. Current anti-influenza measures include vaccines and antivirals (such as Tamiflu^®^ and Relenza^®^), but the former are highly strain-specific and have to be specifically tailored to those viruses which are thought likely to be circulating in the following months, and viruses are gaining resistance to the antivirals. In recent years concerns have been raised about the year to year differences in efficacy of influenza vaccines due to antigenic mismatch with circulating strains and this is compounded by the variation in vaccine efficacy differs between age groups [3,4,5,6].

Defective interfering (DI) viruses arise during the replication of almost all viruses studied to date and are the result of errors during genome replication [7,8]. The DI virus contains a truncated (hence non-infectious or defective) version of the infectious genome that is able to interfere with the production of infectious virus [9]. The position and extent of the genome deletion in the DI virus is highly variable, and there can be a central or a terminal deletion depending on the species of virus involved [7,8]. However, the only defective genomes that qualify as DI virus are those that retain the signals that permit and control genome replication and packaging. DI viruses have a number of interesting biological properties not present in the infectious parent virus: chief among these is the inability to replicate autonomously (i.e., they are defective) and their dependence on an infectious genome in the same cell to provide the functions that have been deleted and are needed for replication. In addition, DI viruses have the ability to become numerically dominant over infectious virus, and lastly they depress the production and yield of infectious virus (i.e., they are interfering) [9,10,11].

The evolutionary significance of DI virus is not known, though they have been detected in patients infected with a range of different viruses [12,13,14,15]. It has been suggested that they play a role in controlling infection and hence in the survival of the host species and ultimately the virus itself, that they result from an inefficient replication process and represent an evolutionarily economic alternative to having more genome to better monitor and correct errors of replication, or that they modulate host immunity in ways that are beneficial to the host [8].

A specific DI influenza A virus, 244/PR8, which is derived from genome segment 1 of influenza A/PR/8/34 (H1N1), has been shown to protect against influenza A virus disease in mice and ferrets following intranasal administration [16,17,18]. In addition, 244/PR8 DI virus stimulates interferon type I and protects in vivo against clinical disease caused by non-influenza A viruses such as influenza B virus and a mouse version of respiratory syncytial virus [19,20]. These data suggest that 244/PR8 DI virus may be useful clinically in preventing disease caused by influenza A and B viruses and other respiratory viruses. To date there have been no studies of the effect of 244/PR8 DI virus in human cells. We describe here the activity of DI influenza A virus in various cells originating from the human respiratory tract, and demonstrate that 244/PR8 is able to significantly reduce the production of infectious influenza A virus in a dose-dependent manner.

## 2. Materials and Methods

### 2.1. Defective Interfering Virus

The influenza virion genome comprises eight segments of single-stranded negative sense RNA. All influenza DI RNAs have a central deletion and retain the termini found in the full-length virion RNA segment [21,22]. We have used reverse genetics to clone a 395-nucleotide DI RNA, called 244/PR8 that is derived from segment 1 of influenza A/PR/8/34 (H1N1) virus [23]. 244/PR8 DI virus was grown in embryonated chicken’s eggs in the presence of infectious “helper” virus to provide the functions missing in the deleted genome segment of the DI virus. The resulting DI virus comprises over 99.9% of the virus present so that it can readily be quantitated using a standard haemagglutinin assay with chicken red blood cells and then converted to micrograms of virus protein (4000 haemagglutination units (HAU) equates to 12 µg). Helper virus infectivity is inactivated by ultraviolet (UV) irradiation at 253.7 nm (0.64 mV/cm^2^) for 40 s, as described previously [23] with the result that the DI virus preparation is non-infectious [16,17,18,19,20,24]. UV irradiation for 8 min completely abrogates the protective ability of the 244/PR8 DI RNA and this provides a material which acts as a control for any effect of virus protein load [23].

### 2.2. Cells

MRC-5 cells are a permanent fibroblast cell line derived from normal human foetal lung tissue. These cells are diploid, have a limited life span of 42–46 cell doublings, and are approved for the preparation of human virus vaccines. Human bronchial epithelial cells (HBEC; TCS Cellworks Ltd., Buckingham, UK) are primary cells isolated from bronchi. The HBEC were grown on a poly-l-lysine coated plastic substrate in the recommended medium (ZHM-1905, TCS Cellworks Ltd., Buckingham, UK) according to the supplier’s instructions, and passaged once before infection.

Primary nasal basal cells were obtained from the respiratory tract of healthy adult human volunteers by brushing the inferior nasal turbinate. None of the subjects were taking medications or had a symptomatic upper respiratory tract infection in the preceding six weeks. All individuals gave their informed consent to be included in the study and all samples were obtained with the individual’s permission. The study was conducted in accordance with the Declaration of Helsinki, and with ethical approval from the University of Leicester Committee for Research Ethics. Cells were washed from the brush with 20 mM 4-(2-hydroxyethyl)-1-piperazineethanesulfonic acid (HEPES)-buffered medium 199 (pH 7.4) (Gibco, Life Technologies, Paisley, UK), and kept at 4 °C overnight. For this study cells were obtained from two different individuals. Basal cells were propagated as previously described [25], and grown to >90% confluence in a T80-collagen-coated flask (Nunclon, Fisher Scientific, Loughborough, UK) containing BEGM™ bronchial epithelial cell growth medium (Lonza, Basel, Switzerland). When confluent, cells were detached with trypsin/ethylenediaminetetraacetic acid (EDTA) solution (Sigma-Aldrich, Gillingham, UK), collected by centrifugation, and seeded at 10^4^ cells/well into a collagen-coated 96-well plate (Corning, Fisher Scientific, Loughborough, UK). These were grown in BEGM™ medium (Lonza) until >80% confluent (4 × 10^4^ cells/well).

### 2.3. Reverse Transcription PCR (RT-PCR) Detection of 244/PR8 DI RNA

Approximately 2 × 10^5^ MRC-5 or HBE cells/well were infected with 5 plaque forming units (pfu)/cell of influenza A/WSN/33 (H1N1) or mock infected with 199 medium for 40 min at room temperature prior to inoculation with serial 10-fold dilutions of 244/PR8 DI virus (9 pg to 9 µg as indicated) for the same time. DI virus was removed, maintenance medium (199 medium containing 2% v/v fetal calf serum) added and incubation continued for 24 h at 33 °C. Medium was removed and cells harvested and lysed in Trizol (Sigma-Aldrich, Gillingham, UK). RNA was extracted and analysed by RT-PCR. 244/PR8 RNA was detected using reverse transcription PCR using the 244/join primer set that specifically anneals to the unique junction region formed as a result of deletion of a central part of the segment 1 sequence and hence detects only 244/PR8 DI RNA generating a 252-nucleotide fragment, as described previously [18]. A 1 kb DNA size ladder (Bioline, London, UK) was used to determine the size of RT-PCR product.

### 2.4. Inhibition of Infectious Virus Multiplication in Primary Nasal Basal Cells by 244/PR8 DI Virus

Nearly confluent basal cells were rinsed with medium and inoculated with 12 pg to 1.2 µg of 244/PR8 DI virus/well or with the same amount of DI virus that had been inactivated by prolonged UV irradiation [23]. Following incubation for 24 h at 37 °C, cells were washed with fresh BEGM™ medium, and infected with influenza A/WSN/33 (10^3^ pfu/well). After incubation for a further 72 h at 37 °C, culture supernatants were collected and assayed for infectivity. Briefly, Madin-Darby canine kidney (MDCK) cells were inoculated with serially diluted virus and incubated for 8 h. Cells were then fixed with 4% paraformaldehyde (Sigma-Aldrich, Gillingham, UK), blocked with 5% milk powder in phosphate-buffered saline (PBS), and incubated with a WSN haemagglutinin (HA)-specific mouse monoclonal antibody (a gift from R. G. Webster, St Jude Children’s Research Hospital, Memphis, TN, USA) in PBS containing 0.1% Tween 20 (PBS-Tween). After removal of the primary antibody, cells were washed with PBS and incubated with a goat anti-mouse IgG antibody (Sigma, Gillingham, UK) coupled to alkaline phosphatase in PBS-Tween. After a further wash with Tris buffered saline (Sigma, Gillingham, UK) to remove unbound material, the cells were incubated with the enzyme substrate (4-nitrophenyl phosphate, Sigma) in diethanolamine buffer solution (Sigma, Gillingham, UK) according to the manufacturer’s instructions, and the absorbance read at 405 nm. The significance of the inhibitory effect of DI virus was determined using a Student’s *t*-test.

Selected basal cell cultures were prepared for immunofluorescence microscopy by fixing overnight with 4% paraformaldehyde (Sigma, Gillingham, UK) in PBS. After blocking with PBS containing 1% bovine serum albumin (BSA, Sigma, Gillingham, UK), cells were stained for WSN HA antigen using a HA-specific mouse monoclonal antibody as above. Unbound antibody was removed and bound antibody was detected using a secondary rabbit anti-mouse immunoglobulin (Ig)G conjugated to Alexa Fluor^®^ 594 (Invitrogen, Life Technologies, Paisley, UK). Nuclei were stained with Hoechst 33258 (4′,6-diamidino-2-phenylindole, DAPI, Invitrogen). Cells were mounted in 50% glycerol in PBS, containing 0.01% *N*-propyl gallate. Low magnification images (20×) were obtained with a Nikon TU1000 fluorescence inverted microscope (Kingston, UK) equipped with a Hamamatsu digital camera (Shizuoka, Japan).

To determine the 50% inhibition level (EC_50_) values, confluent cell cultures in 96 well plates were treated with a 10-fold dilution series of 244/PR8 DI virus or UV inactivated DI virus and incubated for 24 h at 37 °C. The cells were washed with fresh BEGM™ medium, and infected with influenza A/WSN/33 (10^3^ pfu/well). After incubation for a further 72 h at 37 °C, culture supernatants were collected and assayed for infectivity as described above. The data were plotted using Graphpad Prism 6 (GraphPad Software, Inc., La Jolla, CA, USA) using a non-linear fit and normalised to calculate percentage virus replication.

## 3. Results and Discussion

### 3.1. Replication of 244/PR8 DI RNA in MRC-5 and Human Bronchial Epithelial (HBE) Cells

**Figure 1:**
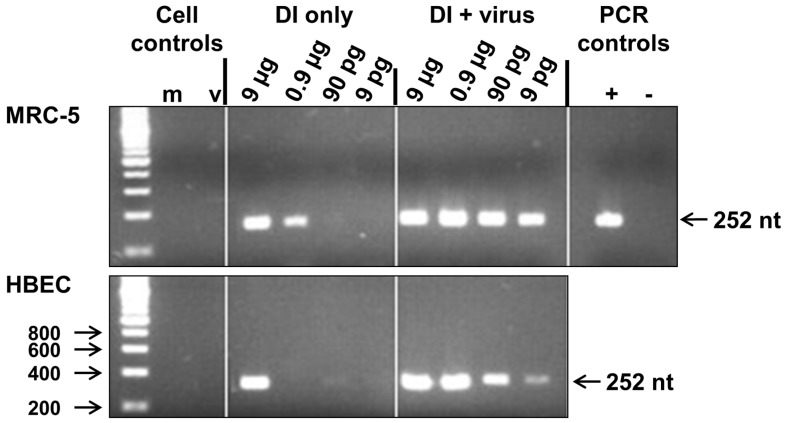
Replication of 244/PR8 defective interfering (DI) RNA in MRC-5 and human bronchial epithelial (HBE) cells in culture. RNA was prepared from cells inoculated with 9 pg, 90 pg, 0.9 µg or 9 µg 244/PR8 DI virus alone or with 244/PR8 DI and infectious A/WSN/33 influenza viruses together, as indicated. 244/PR8 RNA was detected by reverse transcription PCR (RT-PCR) amplification and the 252 nucleotide (nt) product was identified by agarose gel electrophoresis. RNA used for RT-PCR amplification in lane m was extracted from mock infected cells and RNA in lane v was extracted from virus infected cells. Positive and negative PCR controls were generated using purified 244/PR8 DI RNA (+) or by omitting RNA from the RT-PCR reaction (−). A DNA size marker ladder is in the left-hand lane. The size of fragments (nt) is indicated.

It has not previously been shown if influenza 244/PR8 DI RNA can be replicated by infectious virus and exert its interfering activity in human cells derived from the respiratory tract. After treatment of MRC-5 cells with 9 pg, 90 pg, 0.9 µg or 9 µg 244/PR8 DI virus (and no infectious virus), DI RNA was detected at 24 h after administration in cultures that received 900 pg or 9 µg DI virus alone but not in cells that were inoculated lower levels (Figure 1). This suggested that the inoculum DI RNA was retained in cells for at least 24 h and this is consistent with in vivo data from mice showing that treatment with 244/PR8 DI virus prior to infection provides protection from disease [23]. In contrast, in cells inoculated with both DI and infectious WSN virus DI RNA was evident at all dilutions of DI virus, demonstrating that the DI RNA had been replicated by the infectious helper virus (Figure 1). No DI RNA was detected in cells inoculated with infectious virus alone or in mock-inoculated cells. Similar data were obtained from primary HBE cells, though in contrast to the situation with the MRC-5 cells no signal was detected when the cells were treated with 0.9 µg of DI virus alone. The intensity of the DI RNA-derived PCR product detected in virus infected HBE cells treated with 9 pg of DI virus was less than that seen in the corresponding MRC-5 cells. (Figure 1). These data demonstrate that both 244/PR8 DI and infectious influenza A viruses are able to enter human respiratory tract cells and that after entry the 244/PR8 DI RNA is accessible for replication by an infectious influenza A helper virus.

### 3.2. Interference in Primary Human Nasal Basal Cells

**Figure 2:**
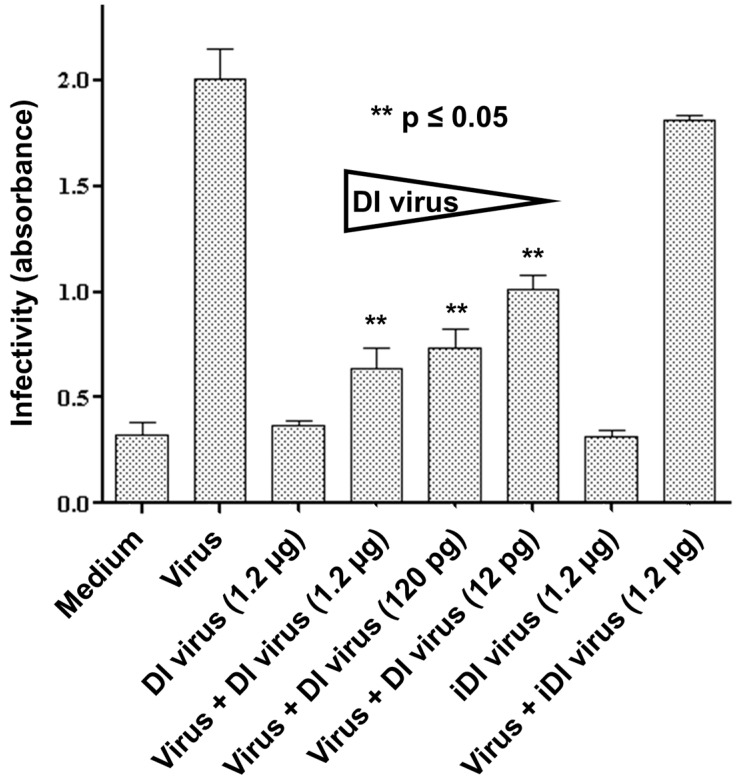
The 244/PR8 DI virus interferes with the multiplication of infectious influenza A/WSN/33 in primary human nasal basal cells. Basal cells were inoculated with 12 pg, 120 pg or 1.2 µg DI virus or 1.2 µg inactivated DI virus (iDI) before infection with influenza A/WSN/33 virus. Culture supernatants were collected and assayed for virus infectivity after 24 h. Infectivity was significantly reduced at all concentrations of DI virus employed (** *p* ≤ 0.05).

Having demonstrated that 244/PR8 DI RNA could be replicated by infectious virus in the MRC-5 cell line and the primary HBE cells, we then examined the ability of 244/PR8 DI virus to interfere with the production of infectious WSN virus in differentiated human basal cells that closely resemble cells of the intact nasal epithelium [25]. No treatment, or treatment with 1.2 µg DI virus alone, or with 1.2 µg UV-inactivated DI virus alone generated no infectious virus, as expected (Figure 2). When 12 pg, 120 pg or 1.2 µg HAU of DI virus were inoculated alongside infectious virus there was a statistically significant reduction in the yield of infectious virus (*p* ≤ 0.05) with all three doses of DI virus compared to the control culture infected with virus alone. Co-infection with UV-inactivated DI virus and infectious virus had no significant effect on the yield of infectious progeny virus (Figure 2). These data show that primary human basal cells support the replication of influenza A virus and that treatment with 244/PR8 DI virus significantly reduces the replication of infectious virus in a dose-dependent manner.

**Figure 3:**
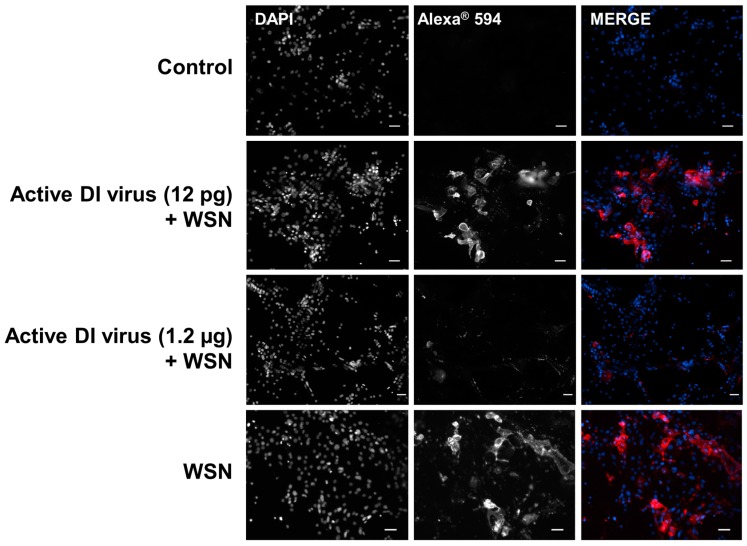
Immunofluorescent detection shows that 244/PR8 DI virus interferes with haemagglutinin (HA) antigen production by influenza A/WSN/33 (WSN) in primary human nasal basal cells. Basal cells were inoculated with bronchial epithelial cell growth BEGM™ medium (control), or 12 pg or 1.2 µg DI virus, as indicated, before infection with influenza A/WSN/33 virus, or infected with WSN alone. Cells were fixed after 24 h and the WSN haemagglutinin protein detected by immunofluorescence. Left panels show cell nuclei stained with 4′,6-diamidino-2-phenylindole (DAPI), middle panels show cells stained with a WSN HA-specific monoclonal antibody and a secondary antibody conjugated with Alexa^®^ 594 (both shown in black and white), and right panels show the merged images in colour. DAPI is blue and Alexa^®^ 594 is red. The images show considerable reduction of the level of influenza A/WSN/33 HA protein in the presence of the higher dose 244/PR8 DI virus. The scale bar indicates 20 µm.

To confirm the effect of the 244/PR8 DI virus on the replication of infectious influenza virus, human basal cells inoculated as above with 12 pg or 1.2 µg of 244/PR8 DI virus and infectious influenza A virus were fixed after removal of the medium at 24 h post infection for the infectivity assay described above. The presence of influenza A/WSN/33 antigen in the fixed cells was detected using a monoclonal antibody specific for the WSN HA protein and a fluorescently labelled secondary antibody (Alexa Fluor^®^ 594; Figure 3). The positive HA fluorescence in cells infected with WSN alone clearly shows the replication of virus in the basal cells. In WSN-infected cells treated with 1.2 µg of 244/PR8 DI virus the level of WSN HA protein detected by fluorescence was significantly reduced at the higher concentration of 244/PR8 DI virus (Figure 3), consistent with the significant reduction in yield of infectious virus when treated with the same amount of 244/PR8 virus shown in Figure 2. The 244/PR8 DI virus is replicated by complementation with influenza A/WSN/33 so that any progeny DI virus is 244/WSN and carries all the WSN antigens. With 12 pg 244/PR8, infectivity was also significantly reduced (Figure 2) although expression of the HA antigen was unaffected (Figure 3). This suggests that DI virus is being synthesized in place of infectious virus. At the higher concentration of 244/PR8 (1.2 µg) little HA antigen can be seen (Figure 3), suggesting that the yield of both DI and infectious virus has been greatly reduced.

The data presented here demonstrate that cloned 244/PR8 DI influenza A virus is capable of delivering DI RNA into three different types of cells derived from the human respiratory tract. This DI RNA was replicated by infectious challenge influenza A virus as has been seen in vivo in mice and ferrets [16,17,18,23]. In primary nasal basal cells the DI RNA was stable for at least 24 h at 33 °C before cells were infected. The yield of challenge virus and its antigen production were reduced in proportion to the dose of applied DI virus, and the calculated EC_50_ in two separate cultures of primary nasal basal cells of 10^−3.04^ and 10^−3.09^ was comparable with the MDCK cell value of 10^−3.27^ (Figure S1). Further data show that primary human basal cells can support the replication of influenza A virus and that treatment with 244/PR8 progressively reduces the replication of infectious virus, HA antigen production, and DI virus itself in a dose-dependent manner. These results represent a significant step forward in validating the use of DI virus as an antiviral in humans.

## Supplementary Materials

The following are available online at www.mdpi.com/1999-4915/8/8/237/s1, Figure S1: Determination of the EC_50_ value for two independent assays of 244/PR8 DI virus in primary nasal basal cells.
