# Supplementary material for: A Defective Interfering Influenza RNA Inhibits Infectious Influenza Virus Replication in Human Respiratory Tract Cells: A Potential New Human Antiviral
Source: Viruses. 2016 Aug 22;8(8):237. doi: 10.3390/v8080237 (PMC4997599; doi:10.3390/v8080237)
Supplement: Supplementary file 1 [file viruses-08-00237-s001.pdf]

Supplementary Materials: A Defective Interfering Influenza RNA Inhibits Infectious Influenza Virus Replication in Human Respiratory Tract Cells: A Potential New Human Antiviral

Claire M. Smith, Paul D. Scott, Christopher O'Callaghan, Andrew J. Easton and Nigel J. Dimmock

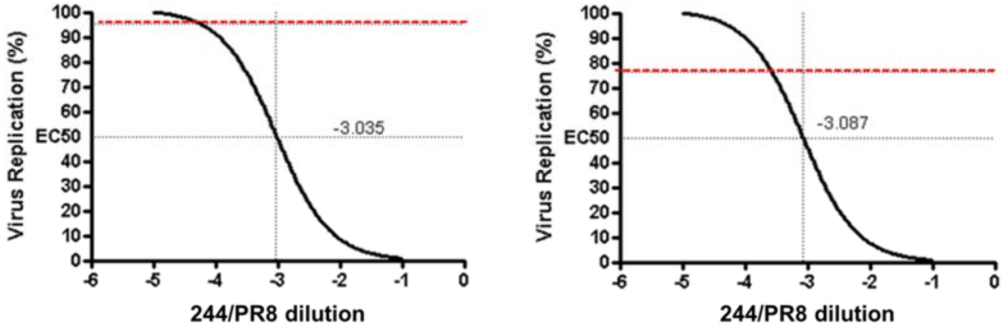

Figure S1. Determination of the EC_{50} value for two independent assays of 244/PR8 DI virus in primary nasal basal cells. Dashed lines show the values for treatment of cells with the control UV inactivated 244/PR8 DI virus in the same assays.

© 2016 by the author; licensee MDPI, Basel, Switzerland. This article is an open access article distributed under the terms and conditions of the Creative Commons Attribution (CC-BY) license (http://creativecommons.org/licenses/by/4.0/).
